# Beverage consumption and individual-level associations in South Korea

**DOI:** 10.1186/1471-2458-13-195

**Published:** 2013-03-06

**Authors:** Euna Han, Tae Hyun Kim, Lisa M Powell

**Affiliations:** 1 Department of Pharmacy, College of Pharmacy and Gachon Institute of Pharmaceutical Sciences, Gachon University, Hambakmeoro 19, Yeonsu-Gu, Incheon, 406-799, South Korea; 2 School of Public Health and Institute of Health Services Research, Yonsei University, 50 Yonsei-ro, Seodaemun-gu, Seoul, South Korea; 3 Department of Health Policy and Administration, School of Public Health and Institute for Health Research and Policy, University of Illinois, 1747 West Roosevelt Road, Chicago, IL, MC 275, USA

**Keywords:** Beverage consumption, Individual-level associations, Two-part regression model, South Korea

## Abstract

**Background:**

Few previous studies investigated consumption distributions of sugar-sweetened beverages (SSBs) over time and individual-level associations in Asia despite the recent interest in SSBs regarding obesity control. This study aimed to provide recent evidence on beverage consumption trends from 2001 to 2009 for overall and subtypes of SSBs and for milk as a comparable healthy beverage in South Korea, as well as associations with individual-level socioeconomic status (SES).

**Methods:**

The Korean National Health and Nutrition Examination Surveys from 2001 to 2009 were used. Consumption prevalence and average caloric intake by SSB type were examined. Associations of SES with consumption were assessed in a multivariate logistic regression model (consumption prevalence) and in a multivariate two-part regression model (overall caloric intake adjusting for consumption probability).

**Results:**

SSB consumption prevalence increased to 38%, 69%, 70%, and 50% by 2009 up from 31%, 66%, 63%, and 32% in 2001 among adolescents, young adults, adults, and the elderly, respectively. Miscellaneous SSBs (sports/energy drinks, coffee/tea products, flavored milk, and others) were the most prevalent and their prevalence increased among adults (from 62% to 69%) and the elderly (from 30% to 47%) between 2001 and 2009. Adolescents consumed the most calories from miscellaneous SSBs among all beverage types although its prevalence was lower than regular soda and milk in both 2001 and 2009. Women (top- income group only) and men in higher income groups showed higher odds of consuming total SSBs (OR = 1.18-1.25), soda (OR = 1.18, men only), fruit drinks (OR = 1.18, the top-income only for both genders), and miscellaneous SSBs (OR = 1.1-1.2). Men with higher-education showed higher odds of total SSB consumption (OR = 1.14-1.20), and all subtypes of SSBs (OR = 1.18, 1.29, 1.19 for soda, fruit drinks, and miscellaneous SSBs, respectively for the top-education group only). There were statistically significant but minimal differences in the overall amount of caloric intake from SSBs by individual SES for both genders.

**Conclusions:**

South Korea is following the global nutrition transition toward greater consumption of SSBs. However, unlike other developed countries, SSB consumption prevalence was higher among high-SES individuals, particularly for fruit drinks and miscellaneous SSBs. Further research is needed to build the international evidence base.

## Background

Obesity has emerged as a global public health concern due to an increase in obesity prevalence in both developing and developed countries [1]. The obesity rate has increased more than three-fold since 1980 worldwide [2]. In the United States, the problem of obesity has been considered epidemic with 72% and 64% of adult men and women estimated to be overweight (body mass index (BMI) ≥ 25 kg/m^2^) and approximately one-third of adults estimated to be obese (BMI ≥ 30 kg/m^2^) in 2008 [3,4]. The obesity prevalence in Korea is not as dramatic as in the United States; however, the prevalence of those overweight reached 31.7% in 2007, an increase from 29.2% in 2005 and 26.0% in 1998. For children and adolescents less than 20 years of age, the prevalence of obesity in Korea doubled over the last decade from 5.8% in 1997 to 10.9% in 2007 [5].

Coincident with the increasing trend of obesity prevalence, previous studies have reported an upward trend in the prevalence of sugar-sweetened beverage (SSB) consumption and the percentage of total energy intake derived from SSBs, including soft drinks and fruit drinks [3,6-9]. The caloric intake of SSBs increased by 135% between 1977 and 2001 in all age groups in the United States [10] although a recent study reported that energy intake from SSBs overall decreased between 1999-2000 and 2007-2008 [11]. SSB sales data also indicated a parallel increase of SSB consumption through to the mid 2000’s with over 14 billion gallons of SSBs being sold in 2008 in the United States [12]. The SSB market has also increased greatly in Korea with total sales of approximately 2.9 billion US dollars in 2008 up from 2.0 billion US dollars in 1999 [13]. SSB consumption has been associated with higher energy intake, reduced consumption of healthier beverages such as milk, reduced nutrient intake, and increased incidence of negative health outcomes such as obesity, type II diabetes [14-17], bone loss [18], heart disease [19], and dental caries [20]. Studies mainly in the U.S. also reported that being non-Hispanic Black [11,21-24], low education [11,25-27], and low income [11,23,24,28] were positively associated with SSB consumption.

Dietary patterns in developing countries such as China and Mexico have experienced a partial transition toward more unhealthy food consumption such as high consumption of fast food and SSBs [29]. However, despite that SSB consumption has been one of the most targeted plausible sources of the obesity epidemic [6] which has been a global phenomenon [2], data for non-US countries are limited and only a few previous studies have explored the nature of SSB consumption outside the U.S. including in Australia and the United Kingdom [25,26,30]. A recent study by Lee and colleagues (2012) explored the consumption trends of food including SSBs between 1998 and 2009 in South Korea, reporting that daily caloric intake from SSBs statistically significantly increased particularly among adolescents aged 13 to 18 and adults aged 19 and 39 years old [31]. Our study builds on the previous literature and investigates the nature of SSB consumption in South Korea, one of the fastest growing developed economies in this millennium [32] with rapid westernization in overall dietary patterns [33]. Understanding the determinants of SSB consumption in a global perspective is important to coordinate global policy measures and to combat obesity. This study provides recent evidence on beverage consumption patterns from 2001 to 2009 for overall and subtypes of SSBs and for milk as a comparable healthy beverage in South Korea. In a multivariate regression framework, we also explore individual-level socioeconomic factors associated with SSB consumption in South Korea.

## Methods

### Data

The study population was comprised of adolescents (12-19 years, N = 3,613), young adults (20-34 years, N = 6,070), adults (35-64 years, N = 14,632), and the elderly (65 years or older, N = 5,102) in the Korean National Health and Nutrition Examination Survey (KNHANES) over nine years from 2001 through 2009 (KNHANES 2001, 2005, 2007, 2008, 2009). The design and data structure of KNHANES are based on the National Health and Nutrition Examination Survey (NHANES) in the United States. These are cross-sectional surveys with study populations from multistage, stratified area probability samples of civilian non-institutionalized Korean households by geographic area, age, and gender groups. Application of complex survey design to adjust for the probability being sampled to each respondent provides nationally representative nutrition and health data with prevalence estimates for nutrition and health status measures [34]. KNHANES addresses a broad range of nutrition- and health-related research questions by providing demographic, socioeconomic, dietary, and health-related information of the respondents. In particular, KNHANES provides detailed dietary information on all foods and beverages consumed in the previous 24 hours (midnight to midnight) through in-person interviews by trained dietary staff in mobile examination centers similar to the U.S. NHANES data.

We included sugar-sweetened soda, fruit drinks, energy and sports drinks (energy/sports drinks), coffee and tea drinks (coffee/tea drinks), flavored milk, and white milk (interchangeably called as milk hereafter). All those beverages except for milk were grouped together as SSBs (i.e., non-alcoholic beverages with added sugar). We also combined energy/sports drinks, coffee/tea drinks, and flavored milk as a category of miscellaneous SSBs. The respondents in the survey reported all food and beverages that were consumed over a 24-hour period, and we aggregated each individual’s relevant consumption records to generate the total energy intake in kilocalories (kcal) from each of the beverage categories.

We drew information on the respondents’ demographic (age, gender, and household size), socioeconomic characteristics (education level, level of per capita household income), and residential area. Age was controlled for in the model as splines with cutoff points for adolescents (aged 12-19 years), young adults (aged 20-34 years), adults (aged 35-64 years), and the elderly (aged 65 or older). Residential area was measured as urban versus non-urban with urban including both large and small cities. Education level was measured as three categories including less than high school, high school, and some college or higher. KNHANES provided information on household income measured as quartiles based on inflation-adjusted per capita household income which we used to classify individuals as being in top, near-top, near-bottom and bottom income groups The summary statistics for our estimation sample are shown in Table 1.

**Table 1:** Weighted summary statistics in the final sample

**Variables**	**Mean (Standard deviation)**	
	**Women**	**Men**
Outcome		
Prevalence of consumption		
Total SSB	0.611	0.641
Soda	0.088	0.105
Fruit drinks	0.080	0.080
Miscellaneous SSBs	0.540	0. 573
Milk	0.289	0.234
Calories consumed (among consumers)		
Total SSB	71.622 (1.108)	99.779 (1.868)
Soda	97.340(3.016)	133.670 (4.368)
Fruit drinks	111.485 (2.745)	129.818 (4.833)
Miscellaneous SSBs	49.404 (0.772)	70.107 (1.458)
Milk	149.902 (2.058)	170.655 (3.269)
Independent variable of interest		
Education: college or more	0.220	0.290
Education: high school graduate	0.351	0.383
Education: < high school (reference group)	0.429	0.327
Household income: top	0.281	0.301
Household income: near-top	0.280	0.292
Household income: near-bottom	0.257	0.250
Household income: bottom (reference group)	0.182	0.157
Covariates		
Age	41.941 (0.231)	39.624 (0.210)
Age spline (adolescents)	7.574 (1.428), [1-8]	7.388 (1.687), [1-8]
Age spline (young adults)	12.100 (5.226), [0-15]	11.555 (5.684), [0-15]
Age spline (adults)	12.172 (11.926), [0-30]	11.586 (11.804), [0-30]
Age spline (elderly)	1.551 (4.160), [0-29]	1.211 (3.526), [0-29]
Household size	3.558(0.021)	3.525 (0.021)
Living in urban	0.814	0.805
Year: 2001	0.072	0.063
Year: 2005	0.226	0.228
Year: 2007	0.231	0.234
Year: 2008	0.234	0.236
Year: 2009	0.237	0.239
N	16,795	12,622

### Analyses

We first examined changes in the consumption prevalence and average caloric intake between 2001 and 2009 by beverage type, including total SSBs, soda, fruit drinks, miscellaneous SSBs, and milk. We then estimated multivariate models by beverage category to assess the associations of individual socioeconomic factors with consumption prevalence and the overall consumption amount adjusting for differential probabilities of consumption by individual factors.

In particular, to assess consumption, we used a two-part regression model to account for a positive mass at zero in the distribution of caloric intake from beverages. For example, 64% of women and 61% of men consumed any SSBs and only 29% of women and 23% of men consumed milk on a given day. For soda and fruit drinks, the respective average prevalence rate per day was 8.8% and 8.0% for women and 10.5% and 8.0% for men (see Table 1). That is, the distribution of caloric intake from beverages was neither discrete nor continuous. The zeros in the distribution of caloric intake were real zeros, neither having the inherent positive values nor being selectively missing. The two-part regression model exploits the fact that the consumption probability splits the model into two parts: part one addresses the discrete feature of the distribution, i.e., the consumption probability for each beverage, and part two accounts for the continuous distribution of caloric intake from the consumed beverages among consumers who consumed more than 0 kcal.

In the first part, the probability of consumption for each beverage category was estimated for the entire sample as shown in *equation* (1), whereas in the second part of the two-part regression model, actual caloric intake from each beverage was estimated conditional on consumption, i.e., among a subset with positive values of caloric intake from each beverage as shown in *equation* (2):

(1) $$\mathrm{Pr}\left(Y>0|X\right)=\Omega \;\left(\mathrm{X\beta},v\right)$$

(2) $$E\left[\left(Y|Y>0,X\right)\right]=\mathrm{X\eta}\;+\;E[\left(\varepsilon |Y>0,X\right)]$$

where Y and X, respectively, represent caloric intake and the set of covariates. The β’s and η’s are parameters to be estimated. *ν* and *ε* are time-varying error terms that were assumed to be normally distributed. The overall consumption-probability-adjusted caloric intake was obtained by multiplying the adjusted consumption probability and adjusted caloric intake from each part of the two-part regression model as in *equation* (3).

(3) $$E\left[\mathrm{overall}\;\mathrm{amount}\;\mathrm{of}\;Y\right]=E[Y\;|Y\;>0,\;X]\times \mathrm{Pr}\left(Y>0|X\right)]$$

Finally, the association of individual characteristics with the overall caloric intake was obtained for the derivatives of the adjusted overall caloric intake as shown in *equation* (4):

(4) $$\begin{array}{ll} \;\frac{\partial E\left[y\right]}{\partial X} & =\frac{\partial \left(\Omega \left(\mathrm{\beta X}\right)\times E\left[y|y>0\right]\right)}{\partial X}\\ & =\left(\mathrm{Pr}\left[y>0\right]\times \frac{\partial E[\;y|y>0]\;}{\partial X}\right)+\left(E\left[y|y>0\right]\times \frac{\partial \mathrm{Pr}\left[y>0\right]\;}{\partial X}\right) \end{array}$$

We applied complex survey design to adjust unequal sampling probabilities and estimated robust standard errors in all models. Survey indicators were controlled for in all models to adjust for a non-linear time trend in beverage consumption. All analyses were run separately by gender. STATA 12.2 was used for all statistical analyses.

## Results

Changes in the prevalence of beverage consumption over time

**Figure 1:**
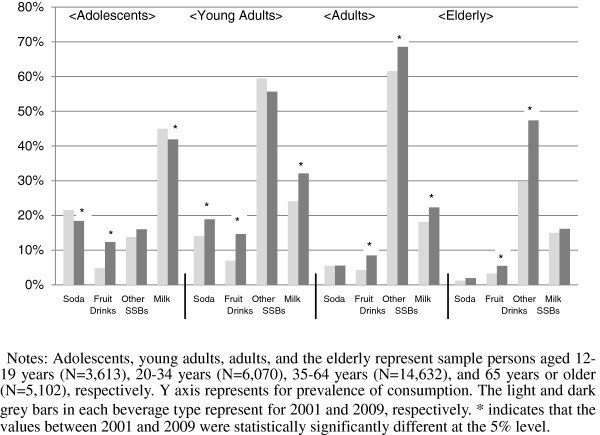
Percent change in the prevalence of consumption between 2001 and 2009 for SSBs and milk.

Miscellaneous SSBs including sports/energy drinks, coffee/tea products, flavored milk and other SSBs were the most prevalent beverage type in both 2001 and 2009 in all age groups except for adolescents with statistically significant increases in prevalence among adults (from 62% to 69%) and the elderly (from 30% to 47%). Milk was the most prevalent beverage consumed for adolescents and the next most consumed in older age groups in both 2001 and 2009 of which the prevalence statistically significantly increased among young adults (from 24% to 32%) and adults (from 18% to 22%). Although the prevalence levels for fruit drinks were not as high as other beverage types, its prevalence increased the most between 2001 and 2009 in all age groups (adolescents 5% to 14%; young adults 7% to 15%; adults 4% to 8%; and the elderly 3% to 6%). The prevalence of soda consumption statistically significantly increased only among young adults (from 14% to 19%), whereas it statistically significantly decreased among adolescents (from 22% to 18%) between 2001 and 2009 (see Figure 1).

### Changes in caloric intake from beverages among consumers over time

**Figure 2:**
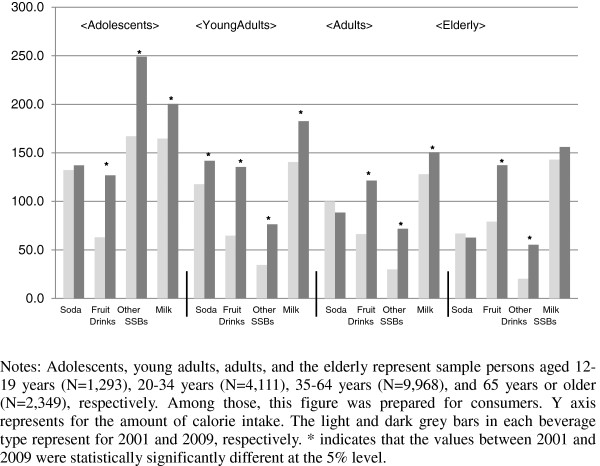
Percent change in energy intake (kcal) between 2001 and 2009 for SSBs and milk among consumers.

The amount of caloric intake conditional on consumption increased for all beverage types in all age groups with the extent of the increase overall being larger for SSBs than milk. Among young adults, adults, and the elderly, the amount of calories consumed from regular soda and fruit drinks were generally much larger than from miscellaneous SSBs despite that they were not as prevalent as miscellaneous SSBs. In contrast, adolescents obtained the most calories from miscellaneous SSB among all beverage types, although its prevalence was lower than regular soda and milk in both 2001 and 2009. Young adults consumed 141.9 kcal of regular soda in 2009, which statistically significantly increased from 117.7 kcal in 2001. The amount of calories from fruit drinks nearly doubled among adolescents (63.0 to 126.8 kcal), young adults (64.7 to 135.5 kcal), adults (66.2 to 121.5 kcal), and the elderly (from 79.1 to 137.3 kcal), surpassing caloric intake from regular soda in 2009 (except for adolescents). Consumers of miscellaneous SSBs doubled their energy intake from it in 2009 (249.0, 76.4, 71.8, and 55.4 kcal for adolescents, young adults, adults, and the elderly, respectively) as compared to 2001 (167.1, 34.4, 29.8, and 20.2 kcal for adolescents, young adults, adults, and the elderly, respectively). The calories consumed from milk were larger than SSBs in both 2001 and 2009 in all age groups and increased statistically significantly among adolescents (164.8 to 200.4 kcal) young adults (140.5 kcal to 182.7 kcal) and adults (127.9 kcal to 150.6 kcal) (see Figure 2).

### Association of individual socioeconomic factors with consumption probability

As shown in Table 2, women in the top-income quartile showed higher odds of consumption for total SSBs (OR = 1.18), fruit drinks (OR = 1.30), and miscellaneous SSBs (OR = 1.13) than women in the bottom-income quartile. However, no statistically significant differences were found in the odds of SSB consumption by education level among women. For milk, both higher-educated and higher-income women were more likely to consume milk than their respective low-SES reference groups (OR = 1.24, 1.16, and 1.13 for the top quartile, near-top quartile and near-bottom quartile, respectively, and OR = 1.52 and 1.18 for college or more education and high school graduate, respectively) (see the upper section of Table 2).

**Table 2:** Factors associated with the consumption probability of SSBs and milk

	**SSB total**		**Soda**		**Fruit drinks**		**Other**		**Milk**	
**Women (N=16,795)**										
Household income:	1.178***		1.031		1.303***		1.130**		1.240***	
High	[1.0671.300]		[0.8921.193]		[1.1491.479]		[1.0251.245]		[1.1131.382]	
Household income:	1.034		0.987		1.140**		1.014		1.156***	
Near-high	[0.9451.130]		[0.8541.139]		[1.0081.290]		[0.9251.110]		[1.0411.284]	
Household income:	1.097**		1.082		1.125*		1.063		1.130**	
Near-low	[1.0051.198]		[0.9341.254]		[0.9911.277]		[0.9731.162]		[1.0241.247]	
Education:	1.026		1.123*		0.988		1.01		1.183***	
High school	[0.9401.119]		[0.9871.279]		[0.8761.115]		[0.9251.103]		[1.0781.298]	
Education:	1.033		1.162*		1.112		1.059		1.519***	
College+	[0.9311.147]		[0.9961.356]		[0.9731.271]		[0.9531.177]		[1.3691.686]	
**Men (N=12,622)**										
Household income:	1.246***		1.180**		1.179**		1.204***		1.204***	
High	[1.1091.400]		[1.0171.369]		[1.0051.383]		[1.0701.356]		[1.0711.355]	
Household income:	1.238***		1.11		1.132		1.279***		1.128**	
Near-high	[1.1061.385]		[0.9561.290]		[0.9641.328]		[1.1481.425]		[1.0021.268]	
Household income:	1.183***		1.116		0.997		1.192***		1.047	
Near-low	[1.0541.328]		[0.9551.304]		[0.8471.173]		[1.0651.333]		[0.9291.181]	
Education:	1.139***		1.099		1.137*		1.152***		1.210***	
High school	[1.0351.252]		[0.9491.272]		[0.9941.300]		[1.0441.271]		[1.0901.343]	
Education:	1.198***		1.184**		1.293***		1.190***		1.480***	
College+	[1.0661.345]		[1.0011.400]		[1.1141.501]		[1.0611.334]		[1.3211.659]	

Notes: Logistic regressions were run for the likelihood of consumption of SSBs and milk. Odds ratios from the logistic regressions were presented. Models also controlled for age as splines for adolescents, young adults, adults, and elderly, household size, residence in urban (with the reference group of rural residence, and survey round as a series of dummy indicators. The reference groups for household income and education group were bottom quartile of household income and less than high school. 95% confidence intervals are in brackets. Standard errors are in parentheses. * *p*< 0.05, ** *p*< 0.01.

Men at higher-income levels showed higher odds of consumption of total SSBs (OR = 1.25, 1.24, 1.18 for the top, near-top, near-bottom income groups, respectively) and miscellaneous SSBs (OR = 1.20, 1.28, 1.19 for the top, near-top, near-bottom income groups, respectively) than those in the bottom income category. Men in the top-income group also showed higher odds of soda and fruit drink consumption (OR = 1.18 for both) than their counterparts in the bottom-income group. Similarly, men with higher levels of education were more likely to consume total SSBs (OR = 1.20 for college or more education and 1.14 for high school graduates) than men with less than high school education. Men with the highest education level (college or more) also were more likely to consume all subtypes of SSBs (OR = 1.18 for soda, 1.29 for fruit drinks, and 1.19 for miscellaneous SSBs) than the lowest education group. For milk, higher-income and higher-educated men showed higher odds of consumption (OR = 1.20 and 1.13 for the top and near-top income groups, respectively, and OR = 1.48 and 1.21 for college or more education and high school graduate, respectively) (see the lower section of Table 2).

### Association of individual socioeconomic factors with the overall amount of caloric intake

Table 3 shows the estimation results from the two-part regression model of the associations of socioeconomic factors with the amount of overall caloric intake adjusting the probability of consumption. Higher-income women consumed statistically significantly more energy per day from total SSBs (+4.0, 3.9, and 3.1 kcal/day for the top, near-top, and near-bottom groups), fruit drinks (+1.6 kcal/day), and miscellaneous SSBs (+2.5, 3.3, and 2.4 kcal/day for the top, near-top, and near-bottom groups) compared to women in the bottom-income group although the differences were minimal. No statistically significant differences by household income level were found for soda intake among women. Women with higher education levels also were found to consume more total SSBs (+3.4 kcal) and all subcategories of SSBs (+2.4 to 2.6 kcal) than their lowest education counterparts. Higher- versus lower-educated women consumed more calories from milk with larger differences for milk than for SSBs; women in the top-income and education groups took in 3.6 and 8.6 kcal/day more than their respective low-SES counterparts (see the upper section of Table 2).

**Table 3:** The individual-level association of selected factors with the consumption of SSBs and milk

**Variables**	**Sugar sweetened beverages**	**Milk**				
	**Total SSBs**		**Soda**	**Fruit drinks**	**Miscellaneous SSB**
**Women**					
Household income:	4.044***	2.434*	1.560**	2.544***	3.625**
High	(1.243)	(1.392)	(0.770)	(0.808)	(1.481)
Household income:	3.928***	1.463	1.125	3.340***	2.251*
Near-high	(1.197)	(1.207)	(0.881)	(0.824)	(1.199)
Household income:	3.120***	1.547	-0.027	2.402***	0.827
Near-low	(0.988)	(1.177)	(0.697)	(0.839)	(1.128)
Education:	2.426**	1.307	1.175*	1.948**	3.730***
College+	(1.032)	(1.065)	(0.680)	(0.764)	(1.220)
Education:	3.339***	2.489**	2.657**	2.385***	8.600***
High school	(1.090)	(1.222)	(1.120)	(0.731)	(1.868)
*N*	*9,985*	*1,289*	*1,262*	*8,578*	*4,473*
**Men**					
Household income:	2.663***	0.377	1.647***	1.529**	7.283***
High	(0.742)	(0.924)	(0.546)	(0.698)	(1.675)
Household income:	0.550	-0.159	0.727*	0.171	4.742***
Near-high	(0.765)	(0.882)	(0.389)	(0.641)	(1.475)
Household income:	1.534**	1.004	0.642*	0.773	3.960**
Near-low	(0.707)	(0.925)	(0.355)	(0.638)	(1.592)
Education:	0.427	1.521*	-0.058	0.128	5.557***
College+	(0.781)	(0.799)	(0.270)	(0.535)	(1.521)
Education:	0.549	2.014**	0.576	0.723	15.515***
High school	(0.816)	(0.959)	(0.375)	(0.695)	(2.344)
*N*	*7,736*	*1,130*	*876*	*6,720*	*2,969*

Two-part regression models were run to estimates the extent of the association of each individual factor with the overall consumption amount in kcal from SSBs and milk when other factors were controlled for. Coefficient estimates from the two-part regression models are presented. Models also controlled for age as splines for adolescents, young adults, adults, and elderly, household size, residence in urban (with the reference group of rural residence, and survey round as a series of dummy indicators. The reference groups for household income and education group were bottom quartile of household income and less than high school. 95% confidence intervals are in brackets. Standard errors are in parentheses. * *p*< 0.05, ** *p*< 0.01.

Men in the near-top income group consumed more total SSBs (+2.3 kcal/day), fruit drinks (+1.6 kcal/day), and miscellaneous SSBs (+1.5 kcal/day). Education was positively associated with the amount of caloric intake only from regular soda (+2.0 kcal/day), although the magnitude of the association was minimal. Men with higher income and higher education also consumed more milk: the top-, near-top, and near-bottom income groups, respectively, consumed 7.3, 4.7, and 4.0 kcal/day more than the bottom-income quartile group; men with college or more and high school educations, respectively, consumed 5.5 and 15.5 kcal/day more than men with less than high school education (see the lower section of Table 2).

## Discussion

Previous studies have documented that the nutrition patterns for major food groups in South Korea have been under a remarkable transition toward more westernized diets over the past few decades [31,35-38]. Our study findings for SSBs based on nationally representative data in South Korea showed that the prevalence of SSB consumption statistically significantly increased among adolescents (31% to 38%), young adults (66% to 69%), adults (63% 70%) and the elderly (31% to 50%) between 2001 and 2009. Our results are comparable to the recent report that between 1998 and 2009 caloric intake from SSBs has increased among adolescents and young to middle-aged adults between 13 and 40 years old [30]. Given that such increases happened in less than a decade, the rate of increase was actually more rapid than that in the U.S. [11,16,39].

However, notably in South Korea, the prevalence of milk consumption also statistically significantly increased between 2001 and 2009 among young adults (24% to 32%) and adults (18% to 22%), although the amount of caloric intake from milk among consumers was less than SSBs. Among adolescents, milk remained the most prevalently consumed beverage in both 2001 and 2009 with no statically significant change between the two time points. Whereas soda has been the most prevalent SSB in the United States in general [11], in South Korea miscellaneous SSBs (sports/energy drinks, coffee/tea products, flavored milk, and others) were the most prevalently consumed and their prevalence statistically significantly increased among adults (62% to 69%) and the elderly (30% to 47%) between 2001 and 2009. Although recent evidence shows that from 1999 to 2008 SSB consumption overall has fallen in the U.S., the prevalence of sports drink consumption increased significantly across all age groups, particularly among adolescents [11]. Our findings are parallel to the recent report that miscellaneous SSBs were the largest source of sugars consumed from processed foods in 2008-2010 for adults in Korea [40], and caloric intake from coffee and tea products increased more than tripled among individuals aged 40-59 between 1998 and 2009 [31].

This present study also found that individuals with higher income (both women and men) and higher education (men only for the prevalence and women only for calorie amount) showed higher odds of SSB consumption and caloric intake from SSBs. However, SES associations with overall caloric intake were quite small. Women and men in the top-income group were more likely to consume SSBs (OR = 1.18 for women and 1.25 for men), although they consumed overall just 4.0 and 2.7 more kcal per day than their counterparts in the bottom-income group. Such findings differ from other studies particularly in the U.S. that have reported higher odds of consumption and higher caloric intake among people in low SES [23,26,27,41]. Our findings imply that increased public health efforts to improve the awareness of the potential negative effects of SSB consumption would be needed in Asian countries including South Korea given the dietary transitions with increasing popularity of western-style meals, particularly among children and adolescents in many developing countries [29].

It is a strength of our study that we estimated individual-level associations for the overall amount of consumption, which accounted for distributional characteristics of the amount of caloric intake as a mix of discrete and continuous forms. Continuous utilization magnitudes were observed only when actual utilization occurred [42,43], and our data showed a sizable segment of the population who actually did not consume the beverages examined in this study. Therefore, a differential consumption probability should be taken into consideration to estimate the overall amount of use in terms of calories.

At the same time, study limitations should be considered in the interpretation of our results. First, our data, KNHANES, had detailed dietary recall information for a 24-hour period similar to the NHANES data in the U.S. However, due to the Korean dining culture in which all main dishes at the same table are typically shared, accurate data on food consumption at the individual level are limited to some extent. KNHANES used individual conversion factors based on age and gender to provide food consumption information at an individual level [44]. The extent to which this conversion represented real individual-level food consumption is not known. At the same time, individually packaged drinks are less likely to be shared, although we could not identify the unit of sales of SSBs in our analyses. Second, we pooled five waves of the KNHANES data including 2001, 2005, 2007, 2008, and 2009, among which seasonally weighted data were not collected for the 2001 and 2005 waves [31]. Third, due to relatively small sample sizes in each survey round, we were unable to estimate differences in individual-level specific associations over time, although we controlled for overall time trends. Fourth, we used stacked cross-sectional data, and thus, we cannot assume causality in interpreting our results. Unobserved individual- or community-level variations such as the regional variation in prices of beverages might have affected the relationship of the individual-level associations with beverage consumption patterns.

SSBs have been reported as one of the key dietary contributors to increased obesity prevalence [45-47]. SSBs, particularly soda, have been at the center of the debate related to obesity control with recent efforts to enact fiscal pricing instruments such as excise taxes on SSBs in the United States and other developed countries [35-37]. South Korea has experienced rapid westernization in overall dietary patterns towards less plant food consumption, more animal food and dietary fat consumption [33,38] despite that some of the traditional dietary patterns have also been retained [31,35,36]. SSBs have not yet been a focus in South Korea regarding obesity control unlike in the U.S. [6,31]. However, this is likely to change given the rapid increase in the prevalence of overweight people in Korea, particularly among children and adolescents [48], and the extant literature reporting the potential negative impact of SSBs on body weight [45] and other related chronic diseases [14-17,19].

Given the high attention to SSBs with regards to obesity control, it is important to assess the nature of SSB consumption in terms of not only time trends but also individual-level associations with the odds of consumption and caloric intake from SSBs in a global perspective. Building on the previous literature, this present study focused on beverage consumption and individual-level association patterns in South Korea. We provided important global evidence on the increase in SSB consumption, particularly for fruit drinks and miscellaneous SSBs. This suggests that South Korea is following the global nutritional transition toward greater consumption of sugars [30,49]. In addition, although our results revealed that higher-SES individuals were more likely to consume SSBs, we found significant but small differences in the extent of caloric intake from SSBs across individual-level SES. Thus, our findings imply that there is a need for policies focusing on all segments of the population to support healthy beverage consumption in South Korea.

## Conclusion

South Korea is following the global nutrition transition toward greater consumption of SSBs. However, the prevalence of SSB consumption was higher among high SES-people unlike other developed countries. Future studies should continue to explore broad international evidence for the determinants of SSB consumption to develop effective policy measures that can minimize any preventable societal costs stemming from the transition and consequential health impacts.

## Abbreviations

BMI: Body Mass Index; KNAHES: Korean National Health and Nutrition Examination Survey; NAHES: National Health and Nutrition Examination Survey; OR: Odds Ratio; SES: Socioeconomic Status; SSB: Sugar Sweetened Beverage

## Competing interests

The authors declare that they have no competing interests.

## Authors’ contributions

EH, THK and LMP contributed to the conception. Han performed the analyses of the data and drafted the paper. Kim conceived the manuscript and led interpretation of the data, and critically revised the paper. Powell helped interpretation of the analysis results and critically revised the paper. All authors read and approved the final version submitted for publication.

## Pre-publication history

The pre-publication history for this paper can be accessed here:

http://www.biomedcentral.com/1471-2458/13/195/prepub
